# Interaction between N6-methyladenosine and autophagy in the regulation of bone and tissue degeneration

**DOI:** 10.3389/fbioe.2022.978283

**Published:** 2022-08-22

**Authors:** Xiaodong Wen, Junhu Wang, Qiong Wang, Peilong Liu, Hongmou Zhao

**Affiliations:** Department of Foot and Ankle Surgery, Honghui Hospital of Xi’an Jiaotong University, Xi’an, China

**Keywords:** degenerative, disease, M6A, autophagy, mRNA, osteoporosis

## Abstract

Bone and tissue degeneration are the most common skeletal disorders that seriously affect people’s quality of life. N6-methyladenosine (m6A) is one of the most common RNA modifications in eukaryotic cells, affecting the alternative splicing, translation, stability and degradation of mRNA. Interestingly, increasing number of evidences have indicated that m6A modification could modulate the expression of autophagy-related (ATG) genes and promote autophagy in the cells. Autophagy is an important process regulating intracellular turnover and is evolutionarily conserved in eukaryotes. Abnormal autophagy results in a variety of diseases, including cardiomyopathy, degenerative disorders, and inflammation. Thus, the interaction between m6A modification and autophagy plays a prominent role in the onset and progression of bone and tissue degeneration. In this review, we summarize the current knowledge related to the effect of m6A modification on autophagy, and introduce the role of the crosstalk between m6A modification and autophagy in bone and tissue degeneration. An in-depth knowledge of the above crosstalk may help to improve our understanding of their effects on bone and tissue degeneration and provide novel insights for the future therapeutics.

## 1 Introduction

Bone and tissue degeneration are common disorders with societal and economic impacts. The most common skeletal degeneration includes osteoporosis, arthritis, and lumbar muscle degeneration, and the common tissue disorders include intervertebral disc degeneration (IVDD) and disc herniation. The above degenerative disorders are characterized by dysfunctional bone- or tissue-derived stem or progenitor cells, and aberrant activation of signaling pathways such as the PTEN, WNT, SIRT1 pathways, and other related signaling pathways (Xiong et al., 2019a; Mi et al., 2020a; Zhang et al., 2023). Currently, researchers in the field of degeneration have focused on discovering the molecular mechanisms mediating the regeneration process and developing new therapeutic strategies for improving the health of patients diagnosed with bone and tissue degeneration (Xiong et al., 2019b; Yu et al., 2020).

N6-methyladenosine (m6A) is one of the most prominent post-transcriptional modifications in eukaryotic mRNA (Mi et al., 2020b; Ren et al., 2022). m6A functionally regulates the eukaryotic transcriptome by influencing mRNA splicing, export, subcellular localization, translation, stability, and decay. Thus, aberrant m6A methylation modulates biological processes and promotes human diseases (Li et al., 2022a). Numerous studies have revealed that m6A methylation plays a crucial role in the regulation of the degeneration process and mediates the occurrence and progression of multiple degeneration-related disorders (Li et al., 2022b; Peng et al., 2022). Intriguingly, prior studies reported that epigenetic modifications including m6A methylation played a prominent role in autophagy regulation (Tang et al., 2022; Wilkinson et al., 2022). Moreover, m6A modification has been reported to directly regulate the expression of autophagy-related (ATG) genes and modulate the cellular autophagy level, and the effects of the m6A methylation on autophagy are dependent on the disease context (Han et al., 2021).

In this review, we aim to summarize the current findings related to the effect of m6A modification on autophagy, and introduce the crosstalk between m6A modification and autophagy with regards to bone and tissue degeneration. An in-depth knowledge of the m6A modification-autophagy axis may expand our understanding of their effects on bone and tissue degeneration and provide novel insights for developing novel therapeutic strategies in the future.

## 2 The current sight of m6A modifications

m6A modification is one of the most abundant modifications in eukaryotic mRNA and is regulated by both m6A methyltransferase and demethylase, which are specifically recognized and bound by the m6A recognition protein (Chen et al., 2022a). m6A modification is dynamically controlled by “writers”, “erasers”, and the “readers”, which are the main methylation-related reading proteins (Table 1) (Shen et al., 2022a). m6A modification affects different stages of mRNA processing including its splicing, nuclear output, stability and translation, and plays a crucial role in gene expression (Zheng et al., 2022). In recent years, owing to the continuous development of methylated RNA immunoprecipitation technology (MeRIP), methylated RNA immunoprecipitation sequencing (MERIP-seq), liquid chromatography-tandem mass spectrometry (LC-MS/MS) and high-throughput sequencing, the methylation modification sites and distribution can be thoroughly identified and analyzed (Sun et al., 2022; Wang et al., 2022). Currently, m6A modifications have been reported to be involved in the modulation of a variety of chronic inflammatory conditions, pathological processes, and metabolism-related diseases (Grenov et al., 2022; Yang et al., 2022).

**TABLE 1:** The common “Writers”, “Erasers”, and “Readers” of m6A methylation.

Category	Genes	Function
Writer	METTL3, METTL14, METTL16, WTAP, KIAA1492,RBM15, HAKAI	Catalyze RNA methylation *in vitro* and *in vivo*
Eraser	FTO, ALKBH5	• Mediate the demethylation of m6A
		•
Reader	YTHDF1, YTHDF2, YTHDF3	Recognize the information of RNA methylation modification and participate in the translation and degradation of downstream RNA

### 2.1 Enzymes involved in m6A modifications

m6A “Writers”, the m6A methyltransferase complex, catalyzes the transfer of methyl groups from S-adenosyl methionine (SAM) to the nitrogen atom at the 6th position of adenine (Yu et al., 2021). m6A writers includes METTL3, METTL14, WTAP, KIAA1429 (VIRMA), RBM15, HAKAI, ZC3H13 (KIAA0853), and METTL16 (Qi et al., 2022). The METTL3-METTL14 methyltransferase complex has been well-documented for its regulatory role in m6A methylation (Liu et al., 2014; Wang et al., 2016). An excellent piece of previous work has suggested that METTL3 primarily functioned as the catalytic core, while METTL14 provided the RNA-binding platform, providing a prominent framework for the functional research of m6A methylation (Wang et al., 2016). Furthermore, another study revealed that METTL3 could interact with the homologous protein METTL14, and form a heterodimer complex, and that the METTL3/METTL14 complex co-catalyzed m6A modification of the target RNA (Liu et al., 2014). In addition to the METTL3-METTL14 methyltransferase complex, m6A-METTL-associated protein complex (MAPC) was also widely reported to be involved in the initiation of m6A modification. The MAPC is mainly formed by the interaction of the junction proteins including WTAP, KIAA1429, RBM15, ZC3H13 and HAKAI (Zhang et al., 2022). Although the RNA splice factor WTAP, has no methyltransferase activity, it acts as a connector protein to recruit the m6A-METTL complex to localize at the nuclear speckle, and thereby regulates the location of m6A modification (Paramasivam et al., 2021). Increasing number of studies have reported new m6A “writers”, such as METTL5, METTL16, and ZCCHC4 (Oerum et al., 2021; Ruszkowska, 2021), suggesting that RNA methyltransferases may include the reported proteins as well as other components, which need to be identified.

m6A “erasers” are able to “elucidate” m6A modifications of the target RNA. Up to date, the fat mass and obesity associated protein (FTO) and ALKB homolog 5 (ALKBH5) are the main components of the m6A demethylases (Shen et al., 2022b). FTO-mediated m6A demethylation has been widely found in multiple biological processes (Li et al., 2017; Wang et al., 2021; Chen et al., 2022b). Li et al. (Li et al., 2017) demonstrated that FTO had a prominent oncogenic role in the development of acute myeloid leukemia (AML) in an m6A-dependent manner. Specifically, FTO promoted leukemic cell transformation and leukemogenesis, and suppressed all-trans-retinoic acid-mediated AML cell differentiation, through the reduction of m6A levels in ASB2 and RARA mRNA transcripts (Li et al., 2017). In bone homeostasis, it was reported that FTO was able to markedly suppress the osteoblastic differentiation of bone marrow derived mesenchymal stem cells (BMSCs) through the demethylation of the m6A modification of runt related transcription factor 2 (Runx2) mRNA (Wang et al., 2021). Intriguingly, Chen et al. (Chen et al., 2022b) found that FTO enhanced the osteogenic differentiation of BMSCs by reducing the stability of PPARG mRNA in an YTHDF1-dependent manner. These seemingly contradictory findings suggest that the role of FTO in regulating bone formation requires further in-depth and accurate investigation. As the second class of m6A “erasers”, ALKBH5 is a Fe^2+^ and α -ketoglutarate-dependent non-heme oxygenase, with a strong ability to demethylate m6A methylation of mRNA (Yu et al., 2022). Up-regulation of ALKBH5 was found to promote demethylate in osteosarcoma cells and suppress cell proliferation and migration, which suggested that ALKBH5 could serve as a potential therapeutic target for treating human osteosarcoma (Yang et al., 2022).

The m6A “readers” can selectively recognize the m6A methylation modifications in the target RNA, and participate in various of stages of RNA metabolism (Zhang and Su, 2022). “Readers” include proteins containing YTH domains (YTHDF1/2/3 and YTHDC1/2), heterogeneous ribonucleoproteins including heterogenous nuclear ribonucleoprotein (HNRNP) C (HNRNPC), G (HNRNPG), and A2B1 (HNRNPA2B1), and insulin-like growth factor 2 binding proteins (IGF2BPs), which are members of a protein family closely associated with several ageing diseases (Li et al., 2022c; Li et al., 2022d). Different “readers” have different cellular localizations and thus perform multiple biological functions (Li et al., 2022d). YTH domain family protein 1 (YTHDF1) initiates RNA translation by interacting with the translation initiation factors and ribosomes, whereas the YTH domain family protein 2 (YTHDF2) selectively binds to m6A modified transcripts and promotes their degradation (Li et al., 2022c). YTHDF1/2 and YTHDF3 play synergistic roles in promoting YTHDF1-mediated translation and suppress YTHDF2-mediated m6A modification (Li et al., 2022d). For example, it was reported that YTHDF2 induced an oncogenic and drug-desensitizing effects in a m6A modification-dependent manner, and could potentially serve as an immune modulating target for intrahepatic cholangiocarcinoma therapy (Huang et al., 2022).

### 2.2 Effect of m6A modifications on mRNA metabolism

mRNA transcription is the process that initiates protein synthesis, and the post-transcriptional modulation of mRNA is controlled by a wide variety of molecular mechanisms. Generally, m6A methylation has been demonstrated to regulate multiple stages of mRNA metabolism (Pan et al., 2021; Wu et al., 2021; Chen et al., 2022c). Chen et al. (Chen et al., 2022c) performed a comprehensive analysis of the relationship between METTL16-mediated m6A methylation and IVDD, and showed that the elevated levels of METTL16 impaired the balance between splicing, maturation, and degradation of MAT2A pre-mRNA and exacerbated IVDD. Furthermore, as the primary demethylase, ALKBH5 was found to inhibit the m6A modification of FOXO3 and enhance its stability (Wu et al., 2021). Here, the anti-tumor effects of ALKBH5 were investigated in-depth, which showed that downregulation of ALKBH5 was associated with poor prognosis in colorectal cancer patients, and revealed that targeting the FOXO3/miR-21/SPRY2 signaling axis could be of therapeutic value for colorectal cancer patients (Wu et al., 2021). Similarly, the critical role of m6A “readers” in mRNA regulation has been well-documented (Pan et al., 2021; Xu et al., 2022). The recent study from Xu et al. (Xu et al., 2022) demonstrated the relationship between YTHDF2 expression and the activation of mTOR/AKT signaling pathway, and showed that the up-regulation of YTHDF2 induced the expression of mTOR mRNA and exacerbated the development of lung squamous cell carcinoma.

### 2.3 Effect of m6A modifications on the maturation of non-coding RNAs

Non-coding RNAs include rRNA, tRNA, snRNA, snoRNA, miRNA, lncRNA, and circRNA. They have a variety of known functions, and at the same time, some of the non-coding RNAs have unknown functions. It was widely reported that m6A methylation is involved in mediating cell proliferation by the induction of miRNA maturation, and the translation and degradation of circRNA, and by altering the stability of lncRNAs (Lin et al., 2020a; Du et al., 2022; Liu and Jiang, 2022; Yan et al., 2022). METTL3-mediated m6A modification has been reported to promote the maturation of miR-146a-5p, which exacerbates the development of bladder cancer (Yan et al., 2022). Furthermore, METTL3 was previously demonstrated to enhance the binding ability of pri-miRNA-589–5p with DGCR8, promoting the malignant progression of hepatoma (Liu and Jiang, 2022). Similarly, a recent study indicated that deoxycholic acid could suppress tumor development by decreasing the maturation of miR-92b-3p in a m6A-dependent manner (Lin et al., 2020a). Moreover, m6A modification was also reported to regulate the translation and degradation of circRNA (Du et al., 2022). circ_0095868 has been identified as a new oncogenic non-coding RNA that was significantly over-expressed in hepatocellular carcinoma (Du et al., 2022). Mechanistically, the m6A “reader” IGF2BP1 was shown to bind to circ_0095868 and promote the stability of circ_0095868 (Du et al., 2022). The role of m6A modification in lncRNA metabolism has also been well-documented (Dai et al., 2022). A recent study from Dai et al. (Dai et al., 2022) reported that METTL16 could target the lncRNA RAB11B-AS1, and impair its stability by elevating the level of m6A. All these findings indicate the strong role of m6A methylation in regulating non-coding RNAs metabolism and function.

## 3 Interaction between m6A and autophagy

### 3.1 Overview of autophagy

**FIGURE 1:**
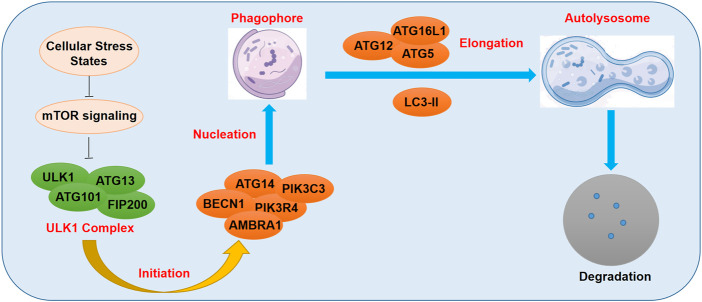
Schematic diagram of m6A methylation regulating autophagy.

Autophagosome is a double-membrane-bound structure which is an important hallmark of the initiation of autophagy, having the ability to bind to the target cellular components (Affortit et al., 2022). Tthe autophagosomes deliver the detrimental cellular components to the lysosome for enzymolysis and degradation (Affortit et al., 2022). A wide range of ATG genes were previously demonstrated to be involved in this regulatory process (Zhang and Klionsky, 2022). Cellular stress states such as oxidative stress injury, hypoxia, and severe nutritional deficiency, were reported to suppress the activity of mTOR complex and activate the Unc-51 like kinase 1/2 (ULK1/2) signaling (Zhang and Klionsky, 2022). The activated ULK1/2 kinase could then induce the formation of FIP200-ATG13 complex, which further increased the level of phosphorylated ULK protein (Zhang and Klionsky, 2022). Subsequently, FIP200-ATG13 complex and the activated phosphorylated ULK proteins recruited more ATG proteins and promoted the formation of double-membrane autophagosomes (Affortit et al., 2022; Deretic and Lazarou, 2022; Zhang and Klionsky, 2022). LC3-II, one of the members of the LC3 family, has been well-documented to promote the formation of autolysosomes through the fusion of the autophagosomes to the lysosomes (Yao et al., 2022), which is a delicately-controlled dynamic process called the autophagy flux (Figure 1). Currently, emerging evidence has indicated that m6A methylation played a prominent role in the modulation of autophagy, and that the m6A-autophagy axis was dependent on the disease context.

### 3.2 Association between m6A and autophagy

m6A modification and the related factors regulate autophagy by modulating ATG expression and autophagy-related signaling pathways (Lin et al., 2020b; Li et al., 2020; Shen et al., 2022c). Li et al. (Li et al., 2020) found that the demethylase ALKBH5, suppressed the level of m6A modification of FIP200 mRNA and induced the expression of FIP200. The FIP200-mediated autophagy flux could subsequently reduce the apoptotic rate of nucleus pulposus cells (NPCs), and thereby ameliorate the development of IVDD (Li et al., 2020). Furthermore, Shen et al. (Shen et al., 2022c) reported that the down-regulation of m6A modification by FTO overexpression suppressed autophagy, which further alleviated liver fibrosis by inducing ferroptosis in hepatic stellate cells. Similarly, METTL3 has been shown to promote the methylation of FOXO3 and induce its binding to YTHDF1 to promote the translation of FOXO3 mRNA, which further inhibited the expression of ATG and suppressed autophagy (Lin et al., 2020b).

It is widely known that the dysregulation of autophagy caused diseases, many of which were closely associated with bone and tissue degeneration. Prior studies have revealed the marked impairment of autophagy in degenerative tissues (Zhong et al., 2022). Enhanced autophagy flux ameliorates oxidative stress, alleviates the progression of degenerative alterations, and enhances the cellular regenerative activity (Yao et al., 2018; Zheng et al., 2021). Increasing number of studies have indicated that m6A-mediated autophagy was involved in the regulation of IVDD and other degenerative diseases (Yao et al., 2018). Taken together, impaired autophagy is associated with the development of degenerative diseases, wherein the m6A autophagy axis plays a critical regulatory role in this process.

## 4 Role of m6A-autophagy axis in the regulation of degenerative diseases

Degenerative changes in NPCs are known to aggravate IVDD, which is the main cause of lower back pain (He et al., 2022). It has been shown that autophagy played a beneficial role in preventing the degeneration of NPCs and markedly ameliorated the progression of IVDD (Li et al., 2021). Similarly, senescence and dysregulation of MSCs are the most prominent reasons for the onset and progression of osteoporosis. Therefore, by enhancing autophagy, cellular senescence and osteogenic differentiation can be significantly rejuvenated.

**FIGURE 2:**
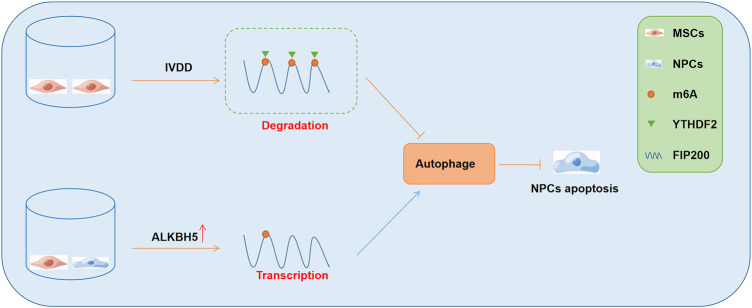
The role of m6A-autophagy axis in the regulation of IVDD development and the underlying mechanisms. NPCs, nucleus pulposus cells; IVDD, intervertebral disc degeneration; MSCs, mesenchymal stem cells.

A recent study showed that the co-culture of MSCs and NPCs elevated the expression of the demethylase ALKBH5, and inhibited m6A modification, which then enhanced the stability of FIP200 and subsequently promoted autophagy (Li et al., 2020). Furthermore, the enhanced autophagy was found to significantly promote the survival of NPCs and ameliorate the development of IVDD (Li et al., 2020). Mechanistically, in the cellular model of IVDD, m6A modification of FIP200 mRNA occurred, and the m6A “reader” YTHDF2 bound to the modified FIP200 transcripts and impaired their stability. However, in the co-culture model with MSCs and NPCs, MSCs significantly promoted the expression of AKLBH5 in the NPCs, which subsequently demethylated the FIP200 mRNAs and prevented their degradation. Consequently, the FIP200-mediated autophagy activity was promoted, which enhanced the survival of NPCs (Figure 2). In the cellular and animal models of osteoarthritis (OA), METTL3 expression was found to be suppressed (He et al., 2022). Furthermore, METTL3 was found to significantly inhibit inflammation-induced apoptosis and autophagy in chondrocytes and was beneficial for delaying the progression of OA (He et al., 2022). Mechanistically, the inhibition of METTL3 decreased the levels of m6A modified BCL-2 and impaired the YTHDF1-mediated mRNA transcription of BCL-2 (He et al., 2022). These findings indicated that METTL3 suppressed apoptosis and autophagy of chondrocytes under inflammatory conditions through the regulation of the m6A-autophagy axis.

## 5 Future perspectives

In summary, the above studies highlight that m6A modification is closely associated with the autophagy process. The up-regulation or suppression of autophagy by m6A methylation mainly depends on the level of m6A, the function of the downstream targets, and the changes in target RNA after methylation. Currently, a large number of studies have demonstrated that m6A modification could regulate the initiation and activation of autophagy by modulating the expression of ULK1, FIP200, and ATG5, ATG7, respectively. Although several researchers have focused on the molecular mechanisms involved, further in-depth studies are urgently needed to elucidate the interaction between m6A modification and autophagy under different pathological conditions.

Currently, the majority of studies have focused on the mediators of m6A. However, the direct regulatory mechanisms of m6A on its downstream targets are still unclear. For example, in hypoxia-induced cancer cells, YTHDF1 was found to enhance autophagy in hepatoma carcinoma cells by enhancing the translation of ATG2A and ATG14 (Li et al., 2021). Whether YTHDF1 could directly affect the expression of ATG2A and ATG14 in an m6A-independent manner and how the changes in m6A regulated ATG2A and ATG14, are topics that need further in-depth research. Additionally, the biggest challenge for revealing the interplay between m6A and autophagy in degenerative diseases is that there are often multiple functions of m6A in different diseases. m6A methylation may act as a “double-edged sword”. For example, m6A not only inhibits the occurrence of IVDD by amelioration of apoptosis in NPCs (Li et al., 2020), but also aggravates apoptosis by inhibiting autophagy (Wang et al., 2020; Chen et al., 2021a; Chen et al., 2021b). The effect of m6A-autophagy axis in various degenerative diseases and the associated mechanisms need further investigation.

The interaction between m6A methylation and autophagy is a attractive topic in cellular biology research; in-depth understanding of the regulators of RNA modification expands the knowledge of the underlying molecular mechanisms. However, more in-depth explorations are required to develop novel therapeutic strategies based on the interaction between m6A methylation and autophagy.
