# Supplementary material for: Interaction between N6-methyladenosine and autophagy in the regulation of bone and tissue degeneration
Source: Front Bioeng Biotechnol. 2022 Aug 22;10:978283. doi: 10.3389/fbioe.2022.978283 (PMC9443517; doi:10.3389/fbioe.2022.978283)
Supplement: Supplementary file 1 [file Table1.DOCX]

**Table 1. The common “Writers”, “Erasers”, and “Readers” of m6A methylation.**

| Category | Genes | Function |
| --- | --- | --- |
| Writer | METTL3, METTL14, METTL16, WTAP, KIAA1492,RBM15, HAKAI | Catalyze RNA methylation *in vitro* and *in vivo* |
| Eraser | FTO, ALKBH5 | Mediate the demethylation of m6A |
| Reader | YTHDF1, YTHDF2, YTHDF3 | Recognize the information of RNA methylation modification and participate in the translation and degradation of downstream RNA |
